# Supplementary material for: Neglected risks in the Chinese insurers’ misconduct caused by digitalization
Source: PLoS One. 2025 Nov 12;20(11):e0336488. doi: 10.1371/journal.pone.0336488 (PMC12611169; doi:10.1371/journal.pone.0336488)
Supplement: Attachment — Submitted filename: Response.docx [file pone.0336488.s003.docx]

**Dear Editors and Reviewers:**

We sincerely thank the editor for providing us with the valuable opportunity to revise the manuscript, and we also deeply appreciate the two reviewers for devoting their time and effort to offering detailed comments. These comments have greatly clarified the direction for revision and helped us improve the quality of the manuscript. We have carefully considered them and revised the manuscript comprehensively. In response to the issues raised by the reviewers, we hereby provide point-by-point replies.

We present the reviewers’ **Comments** in bold black text, our Responses in blue text, and the specific Revision Content in red text. (For the responses and corresponding revisions under each comment, we provide explanations in a point-by-point manner, ensuring that each response corresponds directly to the related revision.) For the Reviewers’ Responses to Questions, our responses and revision details are presented on pages 1-3. For the Review Comments to the Author, our responses and revision details are presented on pages 4-10 (Reviewer 1: pages 4-8; Reviewer 2: pages 9-10). Specifically, these are as follows:

**1. The revision explanation for** **the Reviewer's Responses to Questions**

**Question 1.** Is the manuscript technically sound, and do the data support the conclusions? [The manuscript must describe a technically sound piece of scientific research with data that supports the conclusions. Experiments must have been conducted rigorously, with appropriate controls, replication, and sample sizes. The conclusions must be drawn appropriately based on the data presented.] ***Reviewer #1: Yes, Reviewer #2: Partly.***

**Response:** Thank you for the valuable suggestion. Under your guidance, we systematically reviewed the experimental design. We supplemented the analysis with new robustness test indicators based on higher data collection standards and conducted regression analyses. Additionally, we constructed a multi-period difference-in-differences model based on the "Broadband China" policy to corroborate the research conclusions. These revisions help to avoid inappropriate inferences, thereby strengthening the technical rigor of the study and enhancing the reliability of the conclusions.

**Revision Content:**

(1) The paragraphs 3-6 of the “4.1 Benchmark regression analysis and Robust test”:

“……We utilize text analysis to extract data on the number of penalties from the NFRA ($digital\_count$).

To address potential greenwashing in annual reports, whereby insurers may exaggerate or even fabricate their digital transformation efforts, we train and fine-tune a large language model (LLM) to further filter digital terms. The model is designed to accurately distinguish between action-level and non-action-level statements. For example, a sentence containing a digital transformation term is retained only if it explicitly describes a concrete action undertaken by the firm to advance digital transformation. This approach preserves the applicability of text-based measures while mitigating bias arising from greenwashing, thereby providing a more accurate reflection of firms’ actual progress in digital transformation. Our objective is to minimize the impact of greenwashing, albeit at the risk of underestimating the extent of digital transformation.

The construction proceeds as follows. First, we label all sentences containing digital transformation terms and identify the corresponding firm names and report years. Second, we use a bootstrap sampling method to select 1,000 sentence pairs for manual annotation, and employ a custom-designed prompt to train the LLM. Third, after training, we classify each sentence in the sample according to its semantic level and retain only keywords identified as “action-level” to construct the final digital transformation measure ($digit2$). The LLM is fine-tuned based on Qwen2.5, achieving over 95% classification accuracy on the validation set.

In robustness tests, we use the LLM-based digital transformation measure ($digit2$) and the number of regulatory penalties ($digital\_count$) as alternative independent and dependent variables, respectively……”

(2) Paragraphs 5-8 of the “4.2.1 Multi-Period DID Model”:

“……Second, even when employing large language models to screen digital transformation texts, the risk of greenwashing cannot be fully eliminated. These concerns underscore the necessity of incorporating exogenous shocks rather than relying solely on annual report texts.

To address this, we exploit the timing of digital transformation-related policy announcements as an exogenous shock. Specifically, following Tian and Zhang (2022), we use the “Broadband China” policy as a proxy for the development of the digital economy. The network infrastructure upgrade of the "broadband China" pilot is a widely used exogenous policy shock reflecting digital transformation. Pilot cities and their corresponding implementation years are based on the website of the Ministry of Industry and Information Technology from 2014 to 2016.

Penalty texts contain company, penalty, and region details. We generate a city-year violation variable as the dependent variable and use “Broadband China” pilot cities and time for multi-period DID regression. Policy dummy equals 1 for pilot cities, 0 otherwise; time dummy equals 1 in post-treatment years and 0 otherwise. Figure A in the S1 document shows the parallel trends test. The parallel trends assumption is supported, as the test passes with a two-period lag.

Our findings suggest that policy-induced digital transformation increases insurers’ misconduct, reinforcing our earlier conclusion. Notably, the "Broadband China" policy provides an exogenous shock, affecting the digital infrastructure of local governments rather than the internal transparency of individual companies. If the development of the digital economy promoted by the policy leads to an increase in insurers’ misconduct, and the effects have lagging, long-term, and growth effects (that is, misconduct does not rise immediately after the implementation of the policy, but increases year by year with the impact of insurer digitalization), it is more likely to be a change in corporate behavior itself, rather than simply because digitalization makes misconduct easier to detect. Due to space constraints, the multi-period DID results based on the policy shock are reported in the Table B in the S1 document.”

(3) Column (6) in Table 4:

“Table 4 Benchmark Regression Analysis and Robust Test

| Dependent Variable  Explanatory Variable | $sum\_fine$ | | | | |
| --- | --- | --- | --- | --- | --- |
|  | (1) | (2) | (3) | (4) | (5) |
| $digit1$ | 1.32***  (4.85) | 0.81***  (2.77) | 0.72**  (2.32) | 0.58*  (1.76) | 0.31***  (3.27) |
| Control Variable | No | No | Yes | Yes | Yes |
| Company fixed effect | No | Yes | No | Yes | Yes |
| Year fixed effect | No | Yes | No | Yes | Yes |
| N | 669 | 669 | 557 | 557 | 557 |
| $R^{2}$ | 0.03 | 0.12 | 0.20 | 0.13 | 0.22 |
| Dependent Variable  Explanatory Variable | $sum\_fine$ | | | $sum\_i\_id$ | $sum\_fine'$ |
|  | (6) | (7) | (8) | (9) | (10) |
| $digit1$ |  | 0.58*  (1.76) | 0.19***  (3.03) | 2.37*  (1.80) | 0.44**  (3.08) |
| $digit2$ | 0.34**  (2.10) |  |  |  |  |
| Control Variable | Yes | Yes | Yes | Yes | Yes |
| Company fixed effect | Yes | Yes | Yes | Yes | No |
| Year fixed effect | Yes | Yes | Yes | Yes | No |
| N | 556 | 557 | 557 | 533 | 618 |
| $R^{2}$ | 0.15 | 0.12 | —— | 0.17 | 0.11 |

”

**Question 4.** Is the manuscript presented in an intelligible fashion and written in standard English? [PLOS ONE does not copyedit accepted manuscripts, so the language in submitted articles must be clear, correct, and unambiguous. Any typographical or grammatical errors should be corrected at revision, so please note any specific errors here.] ***Reviewer #1: Yes, Reviewer #2: No.***

**Response and Revision Content:** Thank you for the valuable suggestion. Under your guidance, we carefully refined the language throughout the manuscript, corrected formatting and grammatical errors, and improved the clarity and coherence of expression. We strived to ensure precise and accurate language that conforms to standard academic English norms, thereby enhancing the overall readability of the manuscript.

**Other questions:** The responses from the reviewers are all "Yes".

**2. Explanation of Revisions for the Review Comments to the Author**

**2.1 Explanation of Revisions for Reviewer 1’s Comments**

We greatly appreciate Reviewer 1's recognition of the research content, methodology, and significance of the manuscript, as well as the valuable suggestions for revision. In response to each issue raised, we have made corresponding revisions and provide the following point-by-point replies.

**Comment 1.** The introduction section follows the common presentation style of introducing the research background, identifying literature gaps, posing research questions, and elaborating on research value. However, elaborating on research value seems too long. It is best to concisely point out your research value in the introduction section, so that readers can understand it at a glance and quickly capture your innovation points.

**Response:** Thank you for the valuable suggestion. Under your guidance, we have comprehensively reviewed the content of the “1. Introduction” section, simplifying it as much as possible while ensuring complete expression without altering the original meaning. We paid special attention to condensing the discussion of research value and highlighting the key points of the innovations. In addition, we also streamlined the general descriptions of the research content, methods, and conclusions.

(1) Research value: We reduced the originally lengthy five paragraphs to a more concise three paragraphs.
(2) Innovations: We summarized the key points in the first sentence of each innovation to enable readers to quickly grasp the main contributions.

(3) Research content and methods: We removed expressions that were highly repetitive with later sections and adopted general descriptions for a more concise presentation.

**Revision Content:**

(1) The paragraphs 1-3 of the “1. Introduction”:

“In the global insurance industry, the rapid embrace of digital technologies has significantly reshaped key operational areas, including product marketing, intelligent underwriting, automated claims processing, and fraud detection (Gatteschi et al., 2018; de Andrés & Gené, 2024). While the benefits of technological progress and digitalization are well-documented (Ding et al., 2024; Zhang et al., 2024), their early adoption phase may also open new channels for misconduct and regulatory violations.

Enterprise misconduct is a distinct operational risk, defined as unethical business operations that violate laws or harm stakeholders (Xia et al., 2023). The Chinese insurance market provides a representative case for examination. Regulatory data from China indicate a rise in insurers’ misconduct over the past decade, coinciding with a period of rapid digital transformation in the insurance industry^[1]^. Therefore, the following questions are raised: Are there risks of misconduct in the digital transformation of insurers? Why does the digital transformation of insurers present new challenges in managing misconduct while advancing the industry?

However, existing literature has predominantly emphasized the positive impacts of digitization on market value (Fritzsch et al., 2021), sales innovation (Eckert et al., 2021), product customization (Radwan, 2019), pricing precision (Radwan, 2019), customer service optimization (e Sá et al., 2024), and fraud reduction (Radwan, 2019), with limited attention to potential risks, especially misconduct ones. Consequently, understanding whether digital transformation in the insurance sector is associated with an increase in misconduct contributes to corporate governance and regulatory oversight, especially in light of the growing prevalence of digital technologies among insurers.”

(2) The paragraphs 8-10 of the “1. Introduction”:

“……First, we construct measurement indicators for digital transformation and misconduct through test analysis for insurers……

……Second, we highlight the often-overlooked risks of digital transformation……

……Third, we investigate the mechanism underlying the impact of digital transformation……”

(3) The paragraphs 4-6 of the “1. Introduction”:

“Using data from Chinese insurers between 2010 and 2021, this empirical study is the first to provide evidence on how digital transformation influences insurers’ misconduct and the underlying mechanisms in an evolving insurance market. We compile annual disclosure reports data from 72 insurers in China (the majority of which are non-listed) and construct a dictionary for digital transformation through text analysis. Next, following Raghunandan (2024), we extract penalty amounts and fines imposed on insurers from the violation announcements issued by the regulator^[2]^. Based on the construction of the core variables, we introduce additional control variables and employ firm and year fixed effects to conduct an empirical analysis of the impact of digital transformation on insurers’ misconduct. The results suggest that digital transformation increases insurers’ misconduct, and robustness as well as endogeneity tests confirm the validity of these findings.

We then conduct a heterogeneity analysis by insurer type, capital structure, the implementation of the “Broadband China” policy, and the digital transformation category. The results show that property insurers and those with joint or foreign ownership are more affected by digital transformation regarding misconduct. The “Broadband China” policy accelerates insurers’ digital transformation but also exacerbates misconduct. While basic technological advancements in digital transformation help reduce misconduct, more advanced technology development and application have the opposite effect.

Furthermore, we construct a channel regression model, employing premium income and the cost ratio as proxies for market expansion and financial pressure variables, respectively. The result shows that the digital transformation of insurers increases misconduct by increasing premium income and cost ratio. In addition, we identify moderate variables to explore complementary tests as as evidence of plausible channels. Internally, firms with greater market power face stronger incentives for misconduct, whereas lower handling fees and operating expenses constrain opportunistic behavior. Externally, mature insurance markets curb misconduct, while economic uncertainty exacerbates it. However, changes in regulatory intensity do not significantly mitigate misconduct, suggesting that regulatory improvements alone cannot fully explain the rise in penalties.”

**Comment 2.** Your literature review section seems to be combined with the introduction, followed by the sources of data and the selection of variables. However, a complete causal analysis should be conducted by applying economic theories before officially starting the quantitative analysis to provide theoretical support for your quantitative analysis. But I did not find this part in your article.

**Response:** Thank you for the valuable suggestion. Under your guidance, we have reorganized the literature review, separating it from “1. Introduction” into an independent chapter titled “2. Literature Review and Hypotheses Development.” In this chapter, we supplemented several relevant economic theories, including resource allocation theory, deterrence theory, and information asymmetry theory, to provide a comprehensive causal analysis and theoretical support for the subsequent quantitative analysis.

**Revision Content:**

“**2. Literature Review and Hypotheses Development**

Enterprise misconduct has long been a significant barrier to the sustainability of the insurance industry and the sound functioning of insurers (Schiro, 2006). Scholars have identified a strong connection between insurers' internal management and misconduct (Rizwan, 2019; Ben, 2024; Gunaseelan et al., 2024). Some argue that changes in the external regulatory environment impact insurers’ misconduct (Chen & Hieber, 2016; Srbinoski et al., 2022; Koziol & Kuhn, 2023). Others have explored the consequences of misconduct for insurers and the broader industry (Makau et al., 2021; Talesh & Filho, 2023). Despite extensive research on company management, industry supervision, and economic outcomes, there is a notable gap in the literature regarding the impact of digital transformation on insurers’ misconduct, especially as technologies such as big data, cloud computing, Internet of Things (IoT), blockchain, and artificial intelligence (AI) continue to evolve and integrate into the sector.

To the best of our knowledge, only Wang & Han (2023) and Wang et al. (2024) empirically demonstrate that digital transformation reduces corporate fraud and illegal corporate acts. In the field of insurance, scholars have not discussed this topic in depth. Nevertheless, emerging technologies such as blockchain, cloud computing, and big data enable the automation of insurance transaction processes. On the one hand, this reduces misconduct by insurers, such as misleading sales, by minimizing manual intervention. On the other hand, it also significantly lowers the labor costs associated with insurers' operations. According to resource allocation theory (Maritan & Lee, 2017), this allows insurers to allocate more resources to misconduct management, thereby reducing misconduct risks. Furthermore, the consensus-based features of digital technologies enhances information transparency in the insurance market, making it easier for regulatory authorities to identify and address insurers’ misconduct. In line with deterrence theory (Scholz, 1997), this helps prevent insurers from engaging in improper practices.

Although there are no empirical findings confirming that digital transformation reduces insurers’ misconduct, the aforementioned analysis indicates that digital transformation may contribute to a reduction in such misconduct through multiple channels, including optimizing insurers’ internal management and external environments. Meanwhile, there is no doubt that the research findings of many scholars support the positive impact of the application of digital technology on the management of insurers and the development of the insurance market (Fritzsch et al., 2021; Eckert et al., 2021; Radwan, 2019; e Sá et al., 2024). Therefore, we propose the following hypothesis.

**H0: Digital transformation decreases insurers’ misconduct.**

However, H0 represents the outcome that most scholars expect to see, and it is also a viewpoint supported by related studies in other industries. Nonetheless, it is worth noting that in the insurance industry, the above hypothesis derived from such analyses is partially inconsistent with the actual data (As detailed in footnote ①). Moreover, a few theoretical studies suggest that digitalization introduces novel risks for financial institutions, complicating misconduct management (Jagtiani & John, 2018).

Insurers have made significant progress in product R&D, channel innovation, and regional expansion due to digital transformation. However, insurer management is distinctive in its core operations, relying on the handling of sensitive data and complex risk assessments within a highly regulated environment. From the internal perspective, the substantial resources and investment needed for technological innovation might leave insurers with insufficient means to boost productivity and compliance risk management efficiency, thus facing the “innovator's dilemma” (Christensen, 2015). Studies have indicated that digital transformation can reshape insurers’ business models (Ostrowska, 2021; Srivastava et al., 2024), with these changes potentially resulting in misconduct risks (Ben, 2024). From a regulatory environment perspective, the preliminary implementation of emerging technologies often leads to an uncertain legal and regulatory environment (a regulatory vacuum) (Jagtiani & John, 2018), which can give rise to issues such as privacy leaks, technology misuse, and market unfairness. According to information asymmetry theory (Arrow, 1963), the complexity of digital technology may exacerbate information asymmetry among firms, users, and regulators (Calderon & Ribeiro, 2024), thereby triggering and worsening misconduct.

Accordingly, we further propose the following hypothesis based on the current state of the insurance industry:

**H1: Digital transformation increases insurers’ misconduct.**”

**Comment 3.** There seem to be some grammatical inappropriateness. For example, the article mentions insurer misconduct many times, and it seems more appropriate to change it to insurers' misconduct, and try to maintain consistent expressions for technical terms.

**Response and Revision Content:** Thank you for the valuable suggestion. Under your guidance, we have carefully reviewed the grammar throughout the manuscript, uniformly replaced “insurer misconduct” with “insurers’ misconduct”, and ensured consistency in the usage of other technical terms.

**Comment 4.** Although your robustness test is very rich, it seems to have not touched upon the essence. The digital transformation of insurance companies has extensively used the frequency of annual report words, but the annual report text may have the phenomenon of "greenwashing", leading to measurement bias. It is hoped that a robustness test targeting this issue can be added.

**Response:** Thank you for the valuable suggestion. Under your guidance, we have carefully considered the potential for greenwashing in annual reports and developed corresponding solutions. Specifically, we have made improvements in two main areas: **(1) employing a fine-tuned large language model (LLM) to update the digital transformation measure, and (2) introducing an external digital transformation shock within a difference-in-differences (DID) framework.**

In the existing literature, digital transformation indicators used in regression analyses are generally constructed through three main approaches:

(1) **Objective disclosure data:** Some studies rely on objectively disclosed data, such as the number of robots or the value of purchases of digital equipment, which can directly reflect the degree of digital transformation. However, as most Chinese insurance companies are unlisted, public disclosure of such data is limited, and we do not adopt this approach. Notably, patent disclosure databases allow us to obtain the annual number of patents for each company, which is highly objective. **We have employed this patent data as an instrumental variable for digital transformation in analyzing its effect on corporate misconduct, thereby mitigating potential greenwashing bias**; the regression results are statistically significant.
(2) **Text-based measures:** The dominant approach in recent literature applies text analysis to annual reports to construct digital transformation measures. However, this method lacks accuracy due to the phenomenon of “greenwashing” in annual reports. To enhance the content validity of the measure, **we augment the traditional term-frequency approach with an LLM to perform in-depth semantic parsing and classification, identifying the action level of digital transformation statements.** We then use the LLM-based measure as an alternative variable in robustness checks, and the results are consistent with the baseline regressions, indicating strong robustness.
(3) **Policy-based DID approach:** Other researchers employ a difference-in-differences model, treating policies related to digital transformation as external shocks to distinguish between treatment and control groups and conduct regression analysis. This method does not directly measure digital transformation but instead uses causal identification to estimate its impact on the dependent variable. Even with the LLM-enhanced text-based measure, greenwashing may not be fully eliminated, as firms may exaggerate or embellish their digital transformation efforts without achieving the stated level. Therefore, introducing external shocks as an auxiliary identification tool is necessary. **We employ the “Broadband China” policy as a digital transformation shock in a multi-period DID model, and the empirical results are significant and pass the parallel trend test.**

**Revision Content:** The same as the Revision Content of Question 1 of the Reviewer's Responses to Questions (Pages 1-3).

**Comment 5.** In terms of literature citation, it is advisable to cite literature with the latest year as much as possible, such as Manning, C. D. (1999). Such as the literature is too old, it is recommended that the reference https://doi.org/10.1016/j.envres.2023.118074 and https://doi.org/10.1080/09640568.2024.2311821 these two articles, The journals they are in are all relatively authoritative and updated annually.

**Response:** Thank you for the valuable suggestion. Under your guidance:

(1) We have removed some outdated literature, such as Manning (1999), among others.

(2) We have supplemented several highly authoritative and recent studies, including Ding et al. (2024), Zhang et al. (2024), and others.

**Revision Content:**

(1) The last line of the first paragraph: "3.2.1 Insurers' Digital Transformation":

“…… we construct our digital transformation indicator using a Python-based dictionary approach, which offers greater interpretability (~~Manning,1999;~~ Wu et al., 2021).”

(2) The lines 4-5 of the first paragraph: "1. Introduction":

“……While the benefits of technological progress and digitalization are well-documented (Ding et al., 2024; Zhang et al., 2024), their early ……”

**2.1 The revision explanation for the Comments of Review 2**

We greatly appreciate Reviewer 2's recognition of the research content, methodology, and significance of the manuscript, as well as the valuable suggestions for revision. In response to each issue raised, we have made corresponding revisions and provide the following point-by-point replies.

**Comment 1.** The theoretical framework is underdeveloped and needs a clearer structure and depth.

**Response and Revision Content:** Thank you for the valuable suggestion. Under your guidance, we adjusted the original research framework (1. Introduction, 2. Research Design, 3. Empirical Analysis, 4. Further Mechanisms Analysis, 5. Conclusion and Discussion) to the current one (1.Introduction, 2. Literature Review and Hypotheses Development, 3. Research Design, 4. Empirical Analysis, 5. Further Mechanisms Analysis, 6. Conclusion and Discussion). Among them, we substantially revise the content of "1. Introduction" and supplement that of "2. Literature Review and Hypotheses Development"—efforts that help further enrich and refine our theoretical framework.

**Comment 2.** The way you measure key variables, particularly digital transformation and misconduct, requires stronger justification and validation.

**Response: Thank you for the valuable suggestion. Under your guidance, we have added the following content:**

(1) **Digital transformation**

To address potential greenwashing in annual reports, where insurance companies may exaggerate or misrepresent their digital transformation efforts, **we introduced two additional methods to supplement the empirical tests for the independent variable.** **The first method treats relevant policies as shocks and applies a difference-in-differences model.** Instead of relying on datasets extracted from annual reports, we employ the Broadband China Policy to divide the sample into a treatment group and a control group, thereby facilitating causal identification. **The second method utilizes a large language model (LLM) to identify digital transformation-related vocabulary at the action level.** This vocabulary is then statistically analyzed to reconstruct a digital transformation indicator, which replaces the explanatory variable in robustness tests. The results show that both the difference-in-differences regression and robustness tests produce results consistent with the benchmark regression, maintaining statistical significance.

(2) **Misconduct**

The measurement of misconduct for the dependent variable may be subject to bias. One concern is that regulatory penalties may not be randomly assigned; for example, financial regulators may systematically target larger or riskier firms, or those located in certain regions. Additionally, unobserved non-compliant behavior may result in missing data. We have addressed this situation and proposed solutions in the manuscript. **First, we include company and year fixed effects to reduce bias arising from differences in regulatory intensity across firms. Second, we treat the noncompliance variable as a dummy variable and rerun the regression using a Tobit model, with results showing a significant positive relationship. Third, we reviewed relevant professional literature and found that regulatory violations in information disclosure are a widely used metric for assessing corporate misconduct**. Accordingly, we cited these sources in the manuscript to support our findings.

**Revision Content:** The same as the Revision Content of Question 1 of the Reviewer's Responses to Questions (Pages 1-3).

**Comment 3.** Several interpretations seem one-sided, leaning too heavily on confirming the hypothesis without engaging with alternative explanations.

**Response:** Thank you for the valuable suggestion. Under your guidance, we have substantially revised the content of “1. Introduction” and supplemented the content of “2. Literature Review and Hypotheses Development.” Within this chapter, we review relevant literature and theories on how digital transformation affects insurance companies’ misconduct from two perspectives, and propose Hypothesis 0 and Hypothesis 1, respectively. While the final empirical results support Hypothesis 1, the analytical process of formulating Hypothesis 0 helps to avoid limitations and one-sidedness in the research perspective.

**Revision Content:** The same as the Revision Content of Comment 2 of the revision explanation for the Comments of Review 1 (Pages 6-7).

**Comment 4.** The manuscript would benefit from thorough language editing to improve clarity and professionalism.

**Response and Revision Content:** Thank you for the valuable suggestion. Under your guidance, we have meticulously refined the language throughout the manuscript, corrected formatting and grammatical errors, and optimized the logical flow of expression. We have strived to ensure clear and accurate language that conforms to standard English norms, thereby enhancing the overall readability of the manuscript.

We would like to further thank all editors and reviewers. We have revised the manuscript in accordance with the comments, and hope it now meets the publication requirements. We kindly welcome any further feedback. Should there be any questions, please do not hesitate to contact us—we would be happy to provide explanations and make additional revisions to further improve the manuscript.

Kind regards,

Xiaoqing Guo, Ph.D Candidate
Corresponding Author
Central University of Finance and Economics

1. Since the State Council launched the “Broadband China” plan in 2013, digital transformation in China has accelerated. Concurrently, data from the China Financial Regulatory Administration show that instances of insurers’ misconduct have increased alongside advancements in digital technology. Specifically, there has been a rapid increase in penalties for misconduct since 2017. In 2024, Chinese insurers received 2,762 misconduct penalties, with total fines amounting to 280 million yuan, representing an increase of 137.3% compared with 2017.
2. Using government penalty data to construct the indicator of insurers’ misconduct presents a potential controversy: Does the rise in government-imposed penalties reflect an actual increase in corporate misconduct, or is it merely a result of heightened regulatory scrutiny and an increased likelihood of detection during digitalization? To validate the appropriateness of our variable selection, we employ a range of methods, including the incorporation of interaction terms, the substitution of dependent variables, and quasi-natural experiments. The empirical analysis demonstrates that digital transformation indeed exacerbates insurers’ propensity to engage in misconduct.
