# Supplementary material for: Neglected risks in the Chinese insurers’ misconduct caused by digitalization
Source: PLoS One. 2025 Nov 12;20(11):e0336488. doi: 10.1371/journal.pone.0336488 (PMC12611169; doi:10.1371/journal.pone.0336488)
Supplement: Attachment — Submitted filename: Reponse.docx [file pone.0336488.s004.docx]

**Dear Editors and Reviewers:**

We sincerely thank the Editor for granting us the valuable opportunity to revise the manuscript for the second time. We also deeply appreciate the two reviewers for their continued time and effort: Reviewer 1 has provided constructive minor revision suggestions to refine the work and recommend acceptance after minor revisions, while Reviewer 2 has offered feedback recommending the direct acceptance of the manuscript.

Reviewer 1’s suggestions have effectively helped us identify targeted areas for optimization, which further enhances the rigor and completeness of the manuscript. We have carefully considered each point raised by Reviewer 1 and made focused, minor revisions to the manuscript accordingly. As for Reviewer 2’s recognition and acceptance recommendation, we are equally grateful for their positive evaluation of the work.

In response to the minor revision comments put forward by Reviewer 1 during this second round of revision, we hereby provide point-by-point replies.

We present the reviewers’ **Comments** in bold black text, our Responses in blue text, and the specific Revision Content in red text. (For the responses and corresponding revisions under each comment, we provide explanations in a point-by-point manner, ensuring that each response corresponds directly to the related revision.) Specifically, these are as follows:

**Comment 1.** The mechanism section is not yet sufficiently developed. I recommend strengthening it by drawing on the following five articles: (1)<https://doi.org/10.1016/j.envres.2023.118074>; (2)<https://doi.org/10.1007/s11356-023-30307-z>; (3)<https://doi.org/10.1016/j.esr.2024.101590>; (4)<https://doi.org/10.1016/j.esr.2025.101837>; (5)doi: 10.2166/wp.2025.355.

**Response:** Thank you for the valuable suggestion. Under your guidance, we carefully study these five articles of extremely high academic value and gain great insights into the application of quantile regression and spatial econometric methods. Therefore, to better refine the mechanism section of our paper, we carefully refer to these five articles to optimize the introduction, result presentation, and interpretation of the mechanism analysis.

**Revision Content:**

1) The Second paragraph of the “5.1 Channel Analysis”: Following prior researches (Ding et al., 2024; Chen et al., 2023; Imran et al., 2025; Imran et al., 2025; Ding et al.,2025), we employ quantile regression to better capture the asymmetric effects of digital transformation on corporate misconduct. The results reveal a “risk amplification effect”, whereby digital transformation intensifies misconduct risks among poorly governed firms. It indicates that digital transformation affects firms through specific mechanisms rather than uniformly increasing misconduct, thereby motivating a closer examination of the channels through which digital transformation shapes misconduct.
2) Footnote ⑥: The results show that at lower quantiles (10%-30%), the coefficients are positive but insignificant, indicating limited impact for firms with low misconduct propensity. From the 40th quantile onward, the coefficients become statistically significant (p < 0.05) and exhibit a monotonic upward trend, increasing from 0.86 at the 40th quantile to 1.45 at the 70th, and reaching a maximum of 1.97 at the 90th quantile (p < 0.1).
3) Lines 18-22 of the Fifth paragraph of the “6. Conclusion and Discussion”: In addition, the quantile regression results indicate that for insurers exposed to higher misconduct risks, digital transformation further amplifies propensity for misconduct, suggesting that it may create new opportunities for regulatory arbitrage. Future research could explore this issue in light of this asymmetric effect.

**Comment 2.** The introduction could more clearly articulate the research motivation—for example, why focus on China rather than the Middle East or South America, and why are samples from African regions important?

**Response:** Thank you for the valuable suggestion. Under your guidance, we further supplement our research motivation.

1) We have several reasons for focusing on China rather than other regions: First, in recent years, China’s financial industry generally undergoes digital transformation—especially after the implementation of the Broadband China Policy. At the same time, regulatory data show that as the digital transformation process advances, misconduct in China’s insurance industry is also on the rise. Second, China has unified compliance management standards, and data on insurance companies’ misconduct is accessible through relevant regulatory websites, which supports the conduct of our research.

2) Additionally, our research sample is from China rather than Africa, but the relevant description was not sufficiently clear; we have therefore supplemented and clarified this part as well.

**Revision Content:**

1) Lines 1-10 of the Second paragraph of the “1.Introduction”: “Corporate misconduct constitutes a unique operational risk, characterized by unethical business practices that break laws or harm stakeholders (Xia et al., 2023). Alarmingly, such misconducts exacerbate long-term inefficiencies, which in turn result in regulatory penalties, reputational damage, and market instability (Makau et al., 2021). The Chinese insurance market provides a representative context for analysis, as its regulatory framework is characterized by uniform rules and comprehensive information disclosure, offering consistent violation records and standardized penalty criteria. Consistently, regulatory data from China show an increase in insurers’ misconduct over the past decade—a trend that aligns with the rapid digital transformation of the insurance industry……”

2) Footnote ①: “Since the State Council launched the “Broadband China” plan in 2013, digital transformation in China has accelerated. Concurrently, data from the China Financial Regulatory Administration show that instances of insurers’ misconduct have increased alongside advancements in digital technology. Specifically, there has been a rapid increase in penalties for misconduct since 2017. In 2024, Chinese insurers received 2,762 misconduct penalties, with total fines amounting to 280 million yuan, representing an increase of 137.3% compared with 2017.”

3) First line of the fourth paragraph of the “1.introduction”: “Using data from Chinese insurers between 2010 and 2021, ……”.

**Comment 3.**The implications section could delve deeper into the study’s international relevance—for instance, what lessons might China’s experience offer for resource policies in developing countries across Asia and South America?

**Response:** Thank you for the valuable suggestion. Under your guidance, we have supplemented relevant content in the final chapter of the manuscript. Specifically, we delve into the transferable lessons of China’s experience for resource policies in developing countries, with a focused analysis of nations across Asia and South America. This supplementation strengthens the global perspective of our study.

Revision Content: The Third paragraph of the “6. Conclusion and Discussion”: “China's experience demonstrates that while digitalization often improves insurers’ profitability, it may also create incentives for misconduct that are frequently overlooked. This tension is especially salient for Asian and Latin American countries grappling with analogous challenges of limited insurance coverage and accelerated fintech proliferation. Therefore, understanding the misconduct risk during insurers’ digital transformation is crucial, as it directly affects industry integrity and the sustainability of digital innovation. Existing research largely emphasizes the efficiency and performance gains from digital transformation but pays limited attention to its unintended risks.”

**Comment 4.** The introduction should provide a more in-depth exposition of the research questions.

**Response:** Thank you for the valuable suggestion. Under your guidance:

1) We supplement the research background.

2) Prior to raising the research questions, we further supplement the rationale for doing so.

3) Clearly formulate the research question.

4) After putting forward the research questions, we supplement the value of addressing these questions.

**Revision Content:**

1) The same as the revision content of Comment 2.

2) Lines 10-12 of the Second paragraph of the “1.Introduction”: “This paradox between rapid digitalization and rising misconduct prompts critical questions about the unintended consequences of insurers’ digital transformation……”

3) Lines 12-15 of the Second paragraph of the “1.Introduction”: “……the following questions are raised: Are there risks of misconduct in the digital transformation of insurers? Why does the digital transformation of insurers present new challenges in managing misconduct while advancing the industry?......”

4) Lines 15-17 of the Second paragraph of the “1.Introduction”: “……Answering these questions offers valuable insights for regulators in emerging markets seeking to balance innovation and compliance.”

We would like to further thank all editors and reviewers. We have revised the manuscript in accordance with the comments, and hope it now meets the publication requirements. We kindly welcome any further feedback. Should there be any questions, please do not hesitate to contact us—we would be happy to provide explanations and make additional revisions to further improve the manuscript.

Kind regards,

Xiaoqing Guo, Ph.D Candidate
Corresponding Author
Central University of Finance and Economics
